# Excess weight gain during insulin pump therapy is associated with higher basal insulin doses

**DOI:** 10.1186/s40200-016-0271-5

**Published:** 2016-10-18

**Authors:** Claudia Boucher-Berry, Elaine A. Parton, Ramin Alemzadeh

**Affiliations:** 1 Department of Pediatrics, Section of Pediatric Endocrinology, Children’s Hospital of Illinois, University of Illinois at Chicago, 840 S. Wood Street, M/C 856, 330 CSN, Chicago, IL 60612 USA; 2 Department of Pediatrics, Medical College of Wisconsin, Milwaukee, WI USA; 3 Department of Pediatrics, University of Tennessee Health Science Center, Memphis, TN USA

**Keywords:** Insulin pumps, Obesity, Pediatrics, Type 1 Diabetes, Continuous subcutaneous insulin infusion

## Abstract

**Background:**

While higher total daily dose (TDD) of insulin has been associated with excess weight gain on insulin pump therapy, the role of higher total basal dose (TBD) of insulin on weight gain has not been studied. We evaluated the impact of higher TBD on weight gain in relationship to glycosylated hemoglobin (HbA1c), hypoglycemic episodes, and change in body mass index (BMI) z score in a group of pediatric patients with type 1 diabetes mellitus (T1DM).

**Methods:**

One-year data from 91 (54 Female/37 Male) patients (2.3–17.8 years of age), transitioned from basal-bolus regimen to insulin pump therapy were reviewed. Patients were divided into two groups based on changes in BMI z score: Group 1 (no change or decrease) and Group 2 (increase).

**Results:**

Thirty-three patients in Group 1 and 58 patients in Group 2. The two groups had similar TDD (0.9 ± 0.2 vs. 0.8 ± 0.2 U/kg/day), however Group 1 had a higher bolus: basal insulin ratio (1.8 ± 0.6 vs. 1.5 ± 0.6, *p* < 0.05). While Groups 1 and 2 had similar HbA1c values (7.7 ± 0.7 vs. 7.70 ± 0.6 %; *p* = 0.79) and activity levels (2.2 ± 0.6 vs. 2.2 ± 0.7; *p* = 0.15), Group 2 had higher rates of hypoglycemic episodes (1.0 ± 0.4 vs. 1.5 ± 0.9, *p* < 0.01).

**Conclusion:**

Excess weight gain was associated with lower bolus to basal insulin ratios independent of glycemic control and activity level. Evaluation of bolus and basal insulin doses during insulin therapy is warranted in order to avoid excess weight gain.

**Electronic supplementary material:**

The online version of this article (doi:10.1186/s40200-016-0271-5) contains supplementary material, which is available to authorized users.

## Background

Type 1 Diabetes (T1DM) is an autoimmune disease characterized by pancreatic beta cell destruction leading to decreased insulin secretion [1]. It is the third most prevalent chronic disease of childhood in the US [2, 3], with an estimated prevalence of 1.54 cases/1000 youth [4]. The mainstay of treatment for Type 1 Diabetes is exogenous insulin using a combination of basal and bolus insulin [5–7]. The basal and bolus insulins are responsible for two separate elements of glycemic control. Basal insulin is responsible for the maintenance of euglycemia in the absence of food. Bolus insulin is responsible for metabolizing dietary carbohydrates and for reducing absolute values in blood glucose when in a hyperglycemic state [8].

A well-known disadvantage of exogenous insulin therapy is weight gain associated with insulin administration [9]. Previous studies have shown that using basal-bolus insulin tends to produce less weight gain compared to intermediate acting insulin such as NPH [10, 11]. However, there is still significant weight gain associated with basal-bolus insulin and this can be distressing, especially for adolescents attempting to achieve optimal control. Multiple studies have evaluated the relationship between daily insulin dose and weight gain by examining the factors that play a role in this complex relationship. The United Kingdom Prospective Diabetes Study (UKPDS) showed that total weight gain was associated with total exogenous insulin dose [12]. Patients in the intensive treatment arm of the Diabetes Control and Complications trial (DCCT) showed greater weight gain and a higher average increase in BMI than those that received conventional diabetes treatment [13, 14]. The intensive treatment group gained an average of 3.3 kg compared with 1.2 kg in the conventional group. After 5 years, the increase in BMI remained significantly greater in the intensive group and the differences in weight were still noted 9 years after DCCT [13, 15]. Holl et.al went further and postulated that along with high insulin doses, other factors that predispose to weight gain are initiation of intensified insulin therapy, tight metabolic control and female gender [16]. Holl also suggested that the excessive weight gain may in fact be a direct result of the flexibility of the basal-bolus regimen- allowing for high dietary carbohydrate intake [16].

Looking at the total dose of insulin in comparison to weight gain is only half of the picture. Since the total daily dose (TDD) of insulin is comprised of the basal and the bolus insulin, it is important to elucidate the necessary ratio of basal to bolus insulin that will lead to glycemic control without excessive gains in weight. The standard modality for calculation of basal and bolus insulin dosages uses the basal dose as 50% for the total daily insulin dose [17–20]. The purpose of this study was to assess the relationship between basal and bolus insulin dosages and weight gain. The goal is to answer the question- is it the basal insulin dose or the bolus insulin dose that contributes most to the weight gain associated with insulin therapy.

## Methods

### Study subjects

Data from ninety-one children and adolescents (ages 2.3 – 17.8 years) were collected. All patients were cared for at the Children’s Hospital of Wisconsin Diabetes Center. The participants were converted from basal bolus therapy using injections [once daily glargine and a premeal rapid acting (Aspart) analogue]) to insulin pump therapy. Prior to transitioning to the insulin pump, the families were evaluated by the Diabetes team to assess their ability to successfully transition to insulin pump therapy. Insulin pump education also included insulin adjustment techniques for management of persistent hyperglycemia or hypoglycemia associated with illness and/or exercise.

### Study design

For each patient data were collected at the beginning of the transition period and then 1 year later. Patients were evaluated at quarterly Diabetes clinic visits and the following data were collected: HbA1c, height, weight, BMI, and Tanner stage. Participants were evaluated by their primary endocrinologist for Tanner staging. Total daily insulin dosage per kilogram was calculated and insulin adjustments were made at the discretion of the primary endocrinologist or nurse practitioner to improve Diabetes control.

The average fasting glucose from self-monitored blood glucose (SMBG) documentation was calculated. At every visit, the number and severity of hypoglycemic episodes in the preceding 3 months was determined. The number of severe hypoglycemic episodes was defined as blood glucose less than 50 mg/dl ((<2.8 mmol/l) associated with unconsciousness with or without seizure (expressed as events per 100 patient-years). The frequency of mild to moderate hypoglycemia defined as blood glucose less than 70 mg/dl (<3.9 mmol/l) (with or without behavioral impairment) was expressed as hypoglycemic episodes per week. Hypoglycemia data were recorded at each visit for the preceding 3 months.

Families were routinely asked to report the number of visits to the emergency room and hospital admissions associated with Diabetes that occurred in between outpatient visits. Finally, the families were asked to fill out an activity survey [21] to appropriately describe their child’s activity level. Sedentary activity or a level of “1” was defined as walking/running less than half a mile per day and spending most of their time sitting or watching television. A level of “2” was assigned to anyone who walks between half a mile and 1.5 miles per day and/or spends comparatively more time in active play than reading or watching television. Finally, a level of “3” was assigned to anyone who identified themselves as being active- involved in any exercise program 2–3 times per week. These activity surveys were completed at each visit.

This study was approved by the Institutional Review Board (IRB) of Children’s Hospital of Wisconsin. The IRB approval was obtained for the retrospective review of patients’ clinical charts and therefore, informed consent was not required. The details that might disclose the identity of the subjects under chart review were omitted.

#### Nutritional assessment

All patients and their families were already using carbohydrate counting at baseline. Each subject and his/her family received nutrition and meal planning recommendations and education on the application of carbohydrate counting to flexible insulin regimen, based on established guidelines [22]. Food labels, exchange lists, food models, and restaurant reference guides were used as educational tools. Each patient was evaluated by a nutritionist at quarterly clinic visits. A 24-h dietary recall with assessment of carbohydrate counting skills was performed at each clinic visit. If any issues arose regarding accuracy of implementation of these guidelines, they were addressed at the clinic visit or on a separately scheduled visit with the nutritionist.

### Insulin dosage calculations and HbA1c determination

#### Implementation of insulin pump therapy

Before the initiation of pump therapy, all patients and families were instructed in the mechanics of pump use and wore a demonstrator pump with saline for 3 days. The patient and the family chose the pump brand [MiniMed (Northridge, CA) and Animas (Westchester, PA)]. Patients and families were instructed of risks of pump use, including catheter-site infection, hyperglycemia, hypoglycemia, ketosis, and diabetic ketoacidosis, and potential mechanical problems (i.e., kinked infusion sets, air bubbles, and dislodged tubing), which could interfere with insulin delivery.

On the day prior to pump placement, all patients discontinued their basal insulin the night before and used correction doses of Lispro insulin every 4 h until the morning of pump start. The new TDD was calculated as 75 % of previous TDD (Lispro + basal insulin regimen). Lispro insulin was used in all pumps. Fifty percent of daily dose was used for bolus dosing, and the remainder was used for basal rates. During the first 2 weeks of pump therapy, patients were instructed to perform frequent blood glucose (BG) monitoring (almost every 2 h, including preprandial, postprandial, and overnight levels). Most patients were started on 2 to 3 basal rates and correction doses were continued from the previous insulin regimen. All patients had daily phone contact with a diabetes nurse educator for 7 to 14 days followed by fax or phone contact (2 to 3 times/week) for at least 1 to 2 months.

The HbA1c was determined using the Bayer DCA (Bayer Diagnostics Inc, Tarrytown, NY) 2000 instrument, with a non-diabetic range of 4.5 to 5.7 %.

#### Statistical analysis

The patients were stratified by BMI and BMI z-score. The patients were then divided into two groups; (1) patients whose BMI z-score were less than or the same as the initial BMI z-score from the start of the study and (2) patients whose BMI z-score increased over the last year. The two groups were compared based on their demographics, anthropometrics, pubertal status, insulin dosages and Diabetes control. Calculations were done to assess certain parameters at the beginning and the end of the study. These calculations included; 1) the total insulin dose per kilogram of body weight, 2) basal insulin dose per kilogram of body weight, 3) bolus insulin dose per kg of body weight, 4) the percent of total insulin given as basal and 5) the percent of total insulin given as bolus.

Baseline characteristics were compared with *t*-test and *X* ^2^ analyses. The HbA1c data were analyzed using paired *t*-test and 1 way analysis of variance. The reported values represent the mean ± standard deviation.

## Results

Table 1 summarizes the general characteristics of both groups. There were 33 patients whose BMI z-score had decreased or remained the same (Group 1) and 58 patients whose BMI z-score increased (Group 2) over the course of a year. The average baseline age was 11.9 years with no significant difference in number of female patients between the two groups. Although the subjects in Group 2 were older than the subjects in Group 1 (12.6 ± 3.6 vs. 10.7 ± 4.2), the duration of Diabetes was similar between the two groups (4.0 ± 2.8 vs. 3.9 ± 2.6, respectively). Pubertal development, as assessed by Tanner staging, was similar between the two groups at both points in the study.

**Table 1:** Baseline Demographics

Characteristics	All Patients (*N* = 91)	Group 1 (*N* = 33)	Group 2 (*N* = 58)	*P* value
Mean age (years)	11.9 ± 3.9	10.7 ± 4.2	12.6 ± 3.6	<0.05
% Female	59.3 %	60.6 %	58.6 %	NS
DM duration (years)	3.9 ± 2.6	3.9 ± 2.6	4 ± 2.8	NS
Pre Tanner staging	2.4 ± 1.3	2.2 ± 1.4	2.5 ± 1.3	NS
Post Tanner staging	2.9 ± 1.5	2.5 ± 1.7	3.1 ± 1.4	NS

Data are mean ± SD. Pre-Tanner staging describes the pubertal level at baseline. Post Tanner staging is the pubertal level after one year

Table 2 summarizes the insulin doses as associated with weight and Diabetes control. The “pre” measurements represent the baseline calculations at the start of the study. The “post” measurements represent the calculations performed 1 year after transition to the insulin pump. Data that were collected and are summarized in Table 2 include, BMI z-score,, TDD of insulin, the percentage of the total daily dose that was represented by the basal insulin, bolus/basal insulin ratios, HbA1c fasting blood glucose (FBG), frequency of hypoglycemic episodes over a 2 week period and the activity level. Although the participants in Group 1 had a higher BMI z-score at the beginning of the study, the difference was not found to be significant when compared to the BMI z-score of Group 2. However, after 1 year of pump therapy, the subjects in Group 2 had a significantly higher BMI z-score than the subjects in Group 1 (0.85 ± 0.7 vs. 0.69 ± 0.8). In addition, the ratio of BMI z-score for each group before the study and at 1 year suggests that the increase in BMI z-score for Group 2 was more significant than the decrease of BMI z-score in Group 1. The increase in BMI z-score was not associated with any change in activity level- as the two groups reported similar activity levels at the beginning and at the end of the study.

**Table 2:** Clinical characteristics of Patients before and 1 year after transition to insulin pump

Characteristics	All Patients (*N* = 91)	Group 1 (*N* = 33)	Group 2 (*N* = 58)	*P* value
Pre-BMI z-score	0.6 ± 0.9	1.01 ± 0.7	0.38 ± 0.85	<0.001
Post-BMI z-score	0.80 ± 0.7	0.69 ± 0.8	0.85 ± 0.7	<0.001
Pre-Post BMI z-score	0.79 ± 0.72	-0.31 ± 0.23	0.47 ± 0.43	<0.001
Pre-TDD (U/kg)	0.9 ± 0.2	0.8 ± 0.2	0.9 ± 0.2	NS
Post- TDD (U/kg)	0.9 ± 0.2	0.8 ± 0.2	0.9 ± 0.2	NS
Pre % Basal	43.9 ± 11.6	43.5 ± 12.8	44.3 ± 10.9	NS
Post % Basal	40.7 ± 9.2	36.9 ± 7.6	42.8 ± 9.3	<0.05
Pre-bolus:basal	1.4 ± 0.7	1.5 ± 0.8	1.4 ± 0.7	NS
Post-bolus:basal	1.6 ± 0.7	1.8 ± 0.6	1.5 ± 0.63	<0.05
Pre- HbA1c	8.1 ± 0.9	7.8 ± 0.9	8.3 ± 0.8	<0.01
Post- HbA1c	7.7 ± 0.7^a^	7.7 ± 0.7	7.7 ± 0.6^a^	NS
Pre-FBG	182.3 ± 45.3	179.8 ± 39.9	183.2 ± 48.8	NS
Post-FBG	163.3 ± 32.4	162.5 ± 33.7	163.4 ± 31.7	NS
Pre-hypo/2 week	1.81 + 0.85	1.48 ± 0.75	1.99 ± 0.86	<0.01
Post-hypo/2 week	1.35 + 0.85^b^	1.0 ± 0.4^a^	1.53 ± 0.9^a^	<0.01
Pre-Activity Level	2.0 + 0.66	2.0 + 0.63	2.0 + 0.68	NS
Post-Activity Level	2.2 + 0.69	2.2 + 0.60	2.2 + 0.74	NS

Data are mean ± SD. Total daily dose (TDD), fasting blood glucose (FBG). “Pre” values represent baseline data collected at the beginning of the study per group. “Post” values represent data collected after one year of insulin pump therapy

*p* value, comparison between Group 1 vs. Group 2

^a^ *p* < 0.01, Comparison between pre and post characteristics

^b^ *p* < 0.001, Comparison between pre and post characteristics

Both groups were well-matched at the start of insulin pump therapy in regards to their total daily insulin doses. Although the TDD at the end of the year remained the same between the two groups, Group 2 had a significantly higher percentage of basal insulin than Group 1. In concordance with this, the ratio of bolus to basal insulin at the end of the study was lower in Group 2 than in Group 1 (1.5 ± 0.63 vs 1.8 ± 0.6; *p* < 0.05).

Overall Diabetes control, as defined by HbA1c, were similar between the two groups at the beginning and the end of the year. However, the subjects in Group 2 displayed a lower average HbA1c after 1 year of insulin pump therapy and this was reflected in a decrease in average HbA1c for the entire cohort. Although there was a significant drop in HbA1c for Group 2 from baseline, there was no difference in fasting blood glucose in either group. Finally, at the beginning of the study, the subjects in Group 2 reported significantly more frequent episodes of hypoglycemia than the subjects in Group 1 (1.99 ± 0.86 vs. 1.48 ± 0.75). After 1 year, the average number of episodes of hypoglycemia reported by subjects in Group 2 had decreased significantly, but still remained higher than those reported in Group 1 (1.53 ± 0.9 vs 1.0 ± 0.4).

Table 3 displays the difference in anthropometrics, activity level, insulin requirements and Diabetes control between male and female participants. There was no significant difference in BMI z-score between the sexes. Both groups had similar TDD, percentage of basal insulin and bolus to basal ratios. They both also demonstrated an improvement in HbA1c and in FBG. However, females overall reported a significantly higher number of hypoglycemic episodes as compared to males. At the end of the study, both sexes reported an improvement in the number of hypoglycemic episodes, however, the number in female patients remained higher than that reported by the males.

**Table 3:** Clinical Comparisons between male and female Subjects

Characteristics	Male (*N* = 37)	Female (*N* = 54)	*P*-value
Mean age (years)	11.8 ± 4.0	11.9 ± 3.9	NS
DM duration (years)	4.5 ± 2.6	3.7 ± 2.7	NS
Pre-BMI z-score	0.66 ± 0.88	0.58 ± 0.85	NS
Post-BMI z-score	0.87 ± 0.71	0.74 ± 0.73	NS
Pre-Activity Level	2.0 + 0.68	2.0 + 0.68	NS
Post-Activity Level	2.1 + 0.70	2.2 + 0.67	NS
Pre-TDD (U/kg)	0.82 ± 0.20	0.91 ± 0.26	NS
Post- TDD (U/kg)	0.84 ± 0.20	0.87 ± 0.22	NS
Pre % Basal	43.9 ± 12.6	44.1 ± 10.9	NS
Post % Basal	39.1 ± 8.9	41.7 ± 9.2	NS
Pre-bolus:basal	1.49 ± 0.82	1.43 ± 0.70	NS
Post-bolus:basal	1.69 ± 0.65	1.53 ± 0.65	NS
Pre- HbA1c	8.1 ± 0.8	8.2 ± 0.8	NS
Post- HbA1c	7.6 ± 0.6†	7.8 ± 0.7†	<0.01
Pre-FBG	179.8 ± 39.9	183.2 ± 48.8	NS
Post-FBG	162.5 ± 33.7†	163.4 ± 31.7	<0.05
Pre-hypo/2 week	1.48 ± 0.75	1.99 ± 0.86	<0.01
Post-hypo/2 week	1.0 ± 0.4†	1.53 ± 0.90†	<0.01

Data are mean ± SD. total daily dose (TDD), fasting blood glucose (FBG). Pre” values represent baseline data collected at the beginning of the study per group. “Post” values represent data collected after one year of insulin pump therapy

*p*- value, comparison between male vs. female

†*p* < 0.01, Comparison between pre and post characteristics

## Discussion

In our study, we observed that higher basal doses of insulin in patients treated on an insulin pump regimen can result in significant weight gain. The patients in the study who had a lower bolus:basal ratio experienced an increase in their BMI z-score over 1 year. Previous studies have looked at the overall impact of insulin on weight gain [12, 13, 16, 23] and have shown that intensive insulin therapy is associated with excess weight gain. Instead of looking at the insulin dose as a whole, we chose to investigate the ratio of bolus to basal insulin. Although the total insulin doses per kilogram of body weight remained the same between the two groups over a year duration, the participants in Group 2 saw an increase in their BMI z-score, while the participants in Group 1 experienced no change or saw a decrease in their BMI z-score. The group that experienced the weight gain had a significantly higher basal percentage and a lower bolus to basal ratio than the other cohort. This suggests that the basal insulin is more of a contributor to the weight gain than the total insulin dose.

Multiple studies have been done to investigate the relationship between insulin and weight gain. One mechanism by which basal insulin can lead to increased weight gain is through increased anabolic activity. Insulin acts as an anabolic hormone which reduces lipolysis and protein catabolism and promotes lipogenesis and protein formation. Studies in humans and mice have postulated that the over replacement of insulin in patients with Diabetes produces a general anabolic effect that leads to increased fat accumulation and weight gain [12, 24]. Liu et al explained that it is the physiological fluctuations of insulin which play a critical role in the balance between energy storage and energy consumption. When these fluctuations are disrupted and exposure to insulin becomes continuous- a situation that is synonymous with elevated basal insulin levels- the body balance moves toward energy storage. This leads to a cluster of ectopic fat accumulation and insulin resistance [24].

Another possibility is that the increased basal insulin may result in experienced or perceived hypoglycemia. Increased appetite is an early, involuntary and adaptive response to low blood glucose [25]. These patients may inadvertently be feeding the basal insulin in order to attain euglycemia. In this study, the cohort that gained weight did report more frequent episodes of hypoglycemia than their counterparts. Therefore it is conceivable that the increased insulin is driving the hypoglycemia which is resulting in over eating and excessive caloric intake.

It is important to analyze the ratio of basal to bolus insulin at each clinical visit. The current recommendation is that the basal insulin should be 50% of the total daily dose [17–20]. King et.al, evaluated pump treated patients with Type 1 Diabetes and recommended the basal dose to be calculated as no more than 40 % of the TDD [26]. However in their study, Kuroda et.al, recommended that the basal insulin dose not exceed 30 % of the total daily dose [27]. Our patients were able to maintain good glycemic control with limited weight gain at a basal percentage of 37 %. This suggests that even the 40 % may be an overestimation of what is needed. Interestingly, Kuroda’s study was performed using patients in the hospital setting on a strict diabetic diet, while our study was performed on an outpatient population. Therefore, it is possible to limit the basal insulin and still obtain optimal glycemic control even when the patient is not in a structured environment.

In our population, increasing the amount of basal insulin had the beneficial effect of improving the hemoglobin A1c- but the detrimental effect of increased weight gain. It has been shown that improving Diabetes control decreases the rate of microvascular and macrovascular complications [15]. However, this weight gain can lead to worsening of the cardiovascular risk factors [28]. In the pediatric population, early development of cardiovascular risk factors can progress into development of atherosclerosis as an adult [29–31]. Since cardiovascular disease is the leading cause of death in patients with T1DM [32, 33], it is likely that short term improvement in Diabetes control may lead to long term cardiovascular morbidity and mortality. This further stresses the importance of limiting the basal insulin to help limit the weight gain and the negative consequences associated with weight gain.

By reducing the basal insulin whether through the basal rates in the pump or by reducing the dose of long-acting insulin, it is possible to improve the Diabetes control and minimize weight gain. This goes against the belief that strict Diabetes control will lead to weight gain and may have implications in the management of Type 1 Diabetes, especially in adolescents who are weight-conscious.

## Conclusion

This study was a pilot study designed to look at the relationship between basal and bolus insulin doses with weight gain. Further studies need to be done to evaluate the long term relationship. Our results suggest that keeping the percentage of basal insulin below 40% and encouraging the patient to bolus more frequently may lead to less weight gain with improved Diabetes control.

## Additional file

Additional file 1: Excel- insulin and weight gain. (XLSX 151 kb)

## Abbreviations

BG: Blood glucose
BMI: Body mass index
DCCT: Diabetes Control and Complications Trial
DM: Diabetes Mellitus
FBG: Fasting blood glucose
HbA1c: Hemoglobin A1c
Hypo/2 week: Hypoglycemic episodes in a 2 week period
IRB: Institutional Review Board
NPH: Neutral protamine hagedorn
SMBG: Self monitored blood glucose
T1DM: Type 1 Diabetes Mellitus
TBD: Total basal dose
TDD: Total daily dose
U/kg: Units per kilogram
UKPDS: United Kingdom Prospective Diabetes Study
